# The predictive value of weight evolution in screening for malnutrition in community-dwelling older persons (70+) in Antwerp

**DOI:** 10.1186/s12875-023-02020-w

**Published:** 2023-03-06

**Authors:** Evelien Van Offenwert, Birgitte Schoenmakers

**Affiliations:** Department of Public Health and Primary Care, Academic Centre of General Practice, KU of Leuven, Kapucijnenvoer 7 box 7001, Leuven, 3000 Belgium

**Keywords:** Primary Health Care, Malnutrition, Screening, Body Weight Changes

## Abstract

**Background:**

Experience and research show that screening for malnutrition in primary care mainly takes place by monitoring the weight parameter and that validated screening instruments are hardly used. In this study we examined the effectiveness and predictive value of weight evolution in screening for (risk of) malnutrition in older people living at home, in comparison with a validated screening tool, namely the Mini Nutritional Assessment Short Form (MNA-SF).

**Methods:**

This project was a prospective, longitudinal study with quantitative data that took place in the province of Antwerp (Belgium) from December 2020 until June 2021. The target group of this study consisted of people over 70 living at home who were visited by a home nurse on a regular basis (at least once a month). The outcome measure was the weight evolution over six months compared with the score on the MNA-SF at month six. Weight was measured and recorded once a month during 6 months. At the last weight measurement, the MNA-SF was administered. In order to assess their own nutritional state, three additional questions were asked after taking the MNA-SF.

**Results:**

A total of 143 patients gave consent to participate, of which 89 were women and 54 men. The mean age was 83.7 years (SD6.62) with a range of 70 to 100 years. Based on the MNA-SF score measured after six months, 53.1% (76/143) of participants had a normal nutritional status, 37.8% (54/143) scored risk of malnutrition and 4.9% (7/ 143) was malnourished. In order to detect people with (risk of) malnutrition, a PPV of 78.6%, a NPV of 60.7%, a sensitivity of 19.3% and a specificity of 96.0% were established with a weight evolution of ≥ 5% weight loss at six months. To detect malnutrition, our results showed respectively 33.3%, 98.4%, 71.4% and 92.3%.

**Conclusion:**

In this study, weight evolution has a low sensitivity in screening for (risk of) malnutrition in people over 70 living at home compared to the MNA-SF. However, in order to detect people with malnutrition, this study demonstrated a sensitivity of 71.4% and a specificity of 92.3% for a weight loss of ≥ 5% at six months.

**Supplementary Information:**

The online version contains supplementary material available at 10.1186/s12875-023-02020-w.

## Background

In a world where there is increasing attention for obesity and the associated health risks, it seems paradoxical to highlight malnutrition. However, malnutrition is a heavily underestimated and frequently occurring pathology [1]. In 2013, 7% of the over-70s living at home in Belgium were found to be malnourished [2]. In addition, 29% of this population had a risk of malnutrition [2, 3]. Due to the aging of the population and the increasing survival rate for a number of pathologies, the current prevalence is estimated to be even higher [1, 4]. Furthermore, malnutrition in a home situation will increase due to the decrease in the length of hospitalization and the shift of care to primary care [1].

The negative consequences of malnutrition cannot be underestimated. Studies showed more frequent disease episodes, a slower recovery, a higher risk of falling, a longer hospital stay, more complications, a reduced quality of life and a shorter life expectancy in patients with malnutrition [2, 4]. Depression and self-neglect are considered as consequences of malnutrition [3]. The economic impact of untreated malnutrition in Belgium was estimated at 400 million euros per year in 2005 [5]. Both the biopsychosocial consequences and the economic consequences can be prevented if (the risk of) malnutrition is timely detected and treated [6].

Screening for malnutrition is indispensable but health care providers only recognize half of malnourished patients [2, 7]. Various screening instruments are available to be used in primary care to detect (the risk of) malnutrition in an early stage. In a systematic review, the Mini Nutritional Assessment short form (MNA-SF) is considered the most suitable screening tool for community-dwelling older adults [8]. This test consists of 6 questions, can be performed in 5 min, and has a sensitivity of 98% and a specificity of 94% [9]. Nevertheless, this screening instrument is rarely used in primary care. The tool is insufficiently known by the general practitioner and is seen as time consuming during a routine consultation. Above, there are other practical barriers to pay sufficient attention to prevention [10, 11]. A multidisciplinary approach could make screening for malnutrition more efficient [1, 5].

Practical experience and research show that screening in primary care mainly takes place by monitoring weight and that validated screening instruments are hardly used [5, 10, 11]. However, care providers intuitively indicate that weight loss could be misleading to assess the nutritional state of patients [12]. Therefore, in this study we want to examine how effective and predictive weight evolution is in screening for (risk of) malnutrition in older people living at home, in comparison with a validated screening tool, namely the MNA-SF.

## Method

### Research design

This project was a prospective, longitudinal study with quantitative data. There was no control group. The outcome measure was the weight evolution over six months compared with the score on the MNA-SF at month six.

### Study population

From October to December 2020, home nurse groups in the province of Antwerp (Belgium) were selected by convenience and contacted by telephone to assess their interest in participating in the study. We asked the committed home nurse groups to introduce the study to their patients. The number of participating nurse groups was determined by convenience.

The target group of this study consisted of community-dwelling people older than 70, living in the province of Antwerp (Belgium) and visited by a home nurse on a regular basis (at least once a month). Exclusion criteria that were used were: persons with an estimated life expectancy of less than six months and persons on artificial nutrition. The nurse estimated the life expectancy as determined by the ‘surprise question’ in the Palliative Care Indicator Tool (PICT). If the patients agreed to participate, the researcher and the nurse assessed the eligibility for inclusion.

### Data collection

We provided the home nurses with the necessary information documents and consent forms per patient. We provided the nurses with the questionnaire, in which the MNA-SF and additional questions were described, and the accompanying manual. In patients who met the inclusion criteria and after the informed consent had been signed, we measured and recorded weight once a month during 6 months, in the morning and without clothing. At the last weight measurement, we administered the MNA-SF and classified patients into three categories: normal nutritional status (MNA-SF score of 12–14), risk of malnutrition (score of 8–11) or malnutrition (score of 0–7) [9]. We referred patients with a result on the MNA-SF showing a risk of malnutrition or malnutrition to their GP.

In order to assess own nutritional state, we asked three additional questions immediately after taking the MNA-SF, namely:

- “Do you think you eat well?”
- “Do you still enjoy eating moments?”
- “During the past six months, have there been any situations that caused a sudden weight change? And if so, what caused this?”

Additional demographic data (age, gender, height, place of residence, social situation, multimorbidities and medication) were included in the questionnaire.

### Data analysis and statistics

To test weight evolution as a screening instrument against a validated screening tool, namely the MNA-SF, a sample size of 108 patients was calculated. This was based on the sample size necessary for diagnostic testing, with a significance level (alpha) of 0.05, a strength of 0.80 and a prevalence of (risk of) malnutrition of 36% [2].

All collected data were stored in an Excel file. A descriptive population table was created based on these data. We categorized body mass index (BMI) according to the limit values ​​for people over 65 of the Flemish Institute for Healthy Living [13]. In this study, we defined polypharmacy as 5 different chronic medications, without over the counter supplements [14].

We determined the predictive value of weight evolution, compared to the MNA-SF, using evaluation of the sensitivity and specificity visualized in a Receiver Operating Characteristic (ROC) curve. We calculated the weight evolution by determining the percentage difference between the last and the first weight measurement. The positive predictive value (PPV), negative predictive value (NPV), sensitivity and specificity were calculated for different cut-off values ​​of weight evolution. This was done for screening for the risk of malnutrition and malnutrition, and also separately for the screening for malnutrition.

We performed the statistical analysis in statistical software program R, version 4.0.2.

### Ethics approval

This study was performed in accordance with the Declaration of Helsinki. All participants must give their agreement via informed consent prior to the start of the study. The Ethics Committee of the KU Leuven University Hospitals granted permission for this study on October 15, 2020. (MP016365) All authors gave consent for publication. Participants were informed in advance about publication of the results and consented by signing the informed consent form.

## Results

In the autumn of 2020, we contacted seven different groups of home nurses from the province of Antwerp, Belgium. Each group committed to this study and selected patients who met the inclusion criteria. In total, 143 patients gave their informed consent to participate, of which 89 were women and 54 men. The mean age was 83.7 years (SD 6.62) with a range of 70 to 100 years. (see Table 1).

**Table 1:** Study population description with nutritional status categorization, based on MNA-SF score

	Normal nutritional status (N = 76)	At risk of malnutrition (N = 54)	Malnourished (N = 7)	General population (N = 143)
**Age**				
Mean (SD)	82.8 (6.10)	84.9 (6.59)	87.3 (8.08)	83.7 (6.62)
Median [Min, Max]	83.5 [71.0, 93.0]	85.0 [71.0, 100]	87.0 [73.0, 96.0]	84.0 [70.0, 100]
**Sex**				
Male	29 (38.2%)	19 (35.2%)	4 (57.1%)	54 (37.8%)
Female	47 (61.8%)	35 (64.8%)	3 (42.9%)	89 (62.2%)
**BMI**				
Mean (SD)	29.6 (5.00)	25.3 (6.01)	20.0 (4.02)	27.4 (5.94)
Median [Min, Max]	28.9 [21.2, 44.7]	24.4 [18.9, 48.1]	20.2 [14.5, 25.5]	26.9 [14.5, 48.1]
Missing data	2 (2.6%)	1 (1.9%)	1 (14.3%)	9 (6.3%)
**BMI categorization**				
Underweight (BMI < 23 kg/m²)	5 (6.6%)	22 (40.7%)	5 (71.4%)	32 (22.4%)
Normal (BMI 23-28 kg/m²)	6 (7.9%)	11 (20.4%)	1 (14.3%)	18 (12.6%)
Overweight (BMI 28-33 kg/m²)	30 (39.5%)	6 (11.1%)	0 (0%)	36 (25.2%)
Obesity (BMI > 33 kg/m²)	33 (43.4%)	14 (25.9%)	0 (0%)	48 (33.6%)
Missing data	2 (2.6%)	1 (1.9%)	1 (14.3%)	9 (6.3%)
**Medications (number without over the counter supplements)**				
Mean (SD)	6.75 (3.71)	7.27 (3.97)	5.75 (3.95)	6.94 (3.79)
Median [Min, Max]	6.50 [0, 15.0]	7.00 [0, 18.0]	7.00 [0, 9.00]	7.00 [0, 18.0]
Missing data	4 (5.3%)	5 (9.3%)	3 (42.9%)	17 (11.9%)
**Polypharmacy categorization**				
No polypharmacy (< 5 medications)	19 (25.0%)	13 (24.1%)	1 (14.3%)	33 (23.1%)
Polypharmacy (≥ 5 medications)	53 (69.7%)	36 (66.7%)	3 (42.9%)	93 (65.0%)
Missing data	4 (5.3%)	5 (9.3%)	3 (42.9%)	17 (11.9%)
**Number of multimorbidities, counted by category**				
No multimorbidities	2 (2.6%)	2 (3.7%)	0 (0%)	4 (2.8%)
1 multimorbidity	13 (17.1%)	7 (13.0%)	1 (14.3%)	21 (14.7%)
2 multimorbidities	22 (28.9%)	9 (16.7%)	2 (28.6%)	34 (23.8%)
3 multimorbidities	22 (28.9%)	18 (33.3%)	3 (42.9%)	43 (30.1%)
4 multimorbidities	10 (13.2%)	10 (18.5%)	1 (14.3%)	22 (15.4%)
5 multimorbidities	5 (6.6%)	3 (5.6%)	0 (0%)	8 (5.6%)
6 multimorbidities	0 (0%)	1 (1.9%)	0 (0%)	1 (0.7%)
Missing data	2 (2.6%)	4 (7.4%)	0 (0%)	10 (7.0%)
**Cardiovascular disease**				
Yes	65 (85.5%)	41 (75.9%)	3 (42.9%)	110 (76.9%)
No	9 (11.8%)	9 (16.7%)	4 (57.1%)	23 (16.1%)
Missing data	2 (2.6%)	4 (7.4%)	0 (0%)	10 (7.0%)
**Pneumologic disease**				
Yes	14 (18.4%)	9 (16.7%)	0 (0%)	23 (16.1%)
No	60 (78.9%)	42 (77.8%)	7 (100%)	111 (77.6%)
Missing data	2 (2.6%)	3 (5.6%)	0 (0%)	9 (6.3%)
**Gastrointestinal disease**				
Yes	29 (38.2%)	17 (31.5%)	3 (42.9%)	51 (35.7%)
No	46 (60.5%)	34 (63.0%)	4 (57.1%)	84 (58.7%)
Missing data	1 (1.3%)	3 (5.6%)	0 (0%)	8 (5.6%)
**Endocrinologic disease**				
Yes	31 (40.8%)	19 (35.2%)	1 (14.3%)	51 (35.7%)
No	43 (56.6%)	32 (59.3%)	6 (85.7%)	83 (58.0%)
Missing data	2 (2.6%)	3 (5.6%)	0 (0%)	9 (6.3%)
**Oncologic disease**				
Yes	7 (9.2%)	6 (11.1%)	1 (14.3%)	15 (10.5%)
orthoNo	67 (88.2%)	45 (83.3%)	6 (85.7%)	119 (83.2%)
Missing data	2 (2.6%)	3 (5.6%)	0 (0%)	9 (6.3%)
**Nefrologic disease**				
Yes	11 (14.5%)	4 (7.4%)	3 (42.9%)	18 (12.6%)
No	63 (82.9%)	47 (87.0%)	4 (57.1%)	116 (81.1%)
Missing data	2 (2.6%)	3 (5.6%)	0 (0%)	9 (6.3%)
**Neurologic disease**				
Yes	12 (15.8%)	12 (22.2%)	1 (14.3%)	26 (18.2%)
No	62 (81.6%)	39 (72.2%)	6 (85.7%)	108 (75.5%)
Missing data	2 (2.6%)	3 (5.6%)	0 (0%)	9 (6.3%)
**Orthopedic disease**				
Yes	7 (9.2%)	10 (18.5%)	0 (0%)	17 (11.9%)
No	67 (88.2%)	42 (77.8%)	7 (100%)	118 (82.5%)
Missing data	2 (2.6%)	2 (3.7%)	0 (0%)	8 (5.6%)
**Psychiatric disease**				
Yes	13 (17.1%)	27 (50.0%)	6 (85.7%)	47 (32.9%)
No	62 (81.6%)	26 (48.1%)	1 (14.3%)	90 (62.9%)
Missing data	1 (1.3%)	1 (1.9%)	0 (0%)	6 (4.2%)
**Appetite**				
Yes	70 (92.1%)	48 (88.9%)	3 (42.9%)	122 (85.3%)
No	6 (7.9%)	6 (11.1%)	3 (42.9%)	16 (11.2%)
Missing data	0 (0%)	0 (0%)	1 (14.3%)	5 (3.5%)
**Eating pleasure**				
Yes	70 (92.1%)	45 (83.3%)	3 (42.9%)	119 (83.2%)
No	6 (7.9%)	9 (16.7%)	3 (42.9%)	19 (13.3%)
Missing data	0 (0%)	0 (0%)	1 (14.3%)	5 (3.5%)
**Social, living situation**				
Living alone	41 (53.9%)	25 (46.3%)	5 (71.4%)	73 (51.0%)
Living together	35 (46.1%)	29 (53.7%)	2 (28.6%)	69 (48.3%)
Missing data	0 (0%)	0 (0%)	0 (0%)	1 (0.7%)
**Residence**				
Rural	65 (85.5%)	48 (88.9%)	7 (100%)	123 (86.0%)
Urban	11 (14.5%)	6 (11.1%)	0 (0%)	19 (13.3%)
Missing data	0 (0%)	0 (0%)	0 (0%)	1 (0.7%)
**Home nursing**				
Private groups	53 (69.7%)	37 (68.5%)	6 (85.7%)	102 (71.3%)
“Wit-Gele Kruis”	23 (30.3%)	17 (31.5%)	1 (14.3%)	41 (28.7%)

*MNA-SF: mini nutritional assessment short form; SD: standard deviation; Min: minimum; Max: maximum; BMI: body mass index*

Based on the MNA-SF score measured after six months, 53.1% (76/143) of participants had a normal nutritional status, 37.8% (54/143) was at risk of malnutrition and 4.9% (7/ 143) was malnourished. The MNA-SF could not be administered to six participants because of death, hospitalization or admission to a residential care center. Of the participants with (risk of) malnutrition, 44.3% (27/61) had a BMI < 23 kg/m², which is defined as underweight for older people [13]. From the participants with a normal score on the MNA-SF was 6.6% (5/76) underweight. The malnourished population had a mean BMI of 20.0 (SD 4.02), compared to 29.6 (SD 5.00) in the population with a normal score on the MNA-SF.

Of the total population, 32.9% (47/143) had a psychiatric multimorbidity. 70.2% (33/47) of people with psychiatric multimorbidity had (risk of) malnutrition. With regard to own nutritional assessment, 83.6% (51/61) of the participants with (risk of) malnutrition believed that they were well nourished and 78.7% (48/61) of this group indicated that they enjoyed eating moments, compared to respectively 92.1% (70/76) and 92.1% (70/76) of the participants with a normal score on the MNA-SF. (see Table 1).

**Figure 1:**
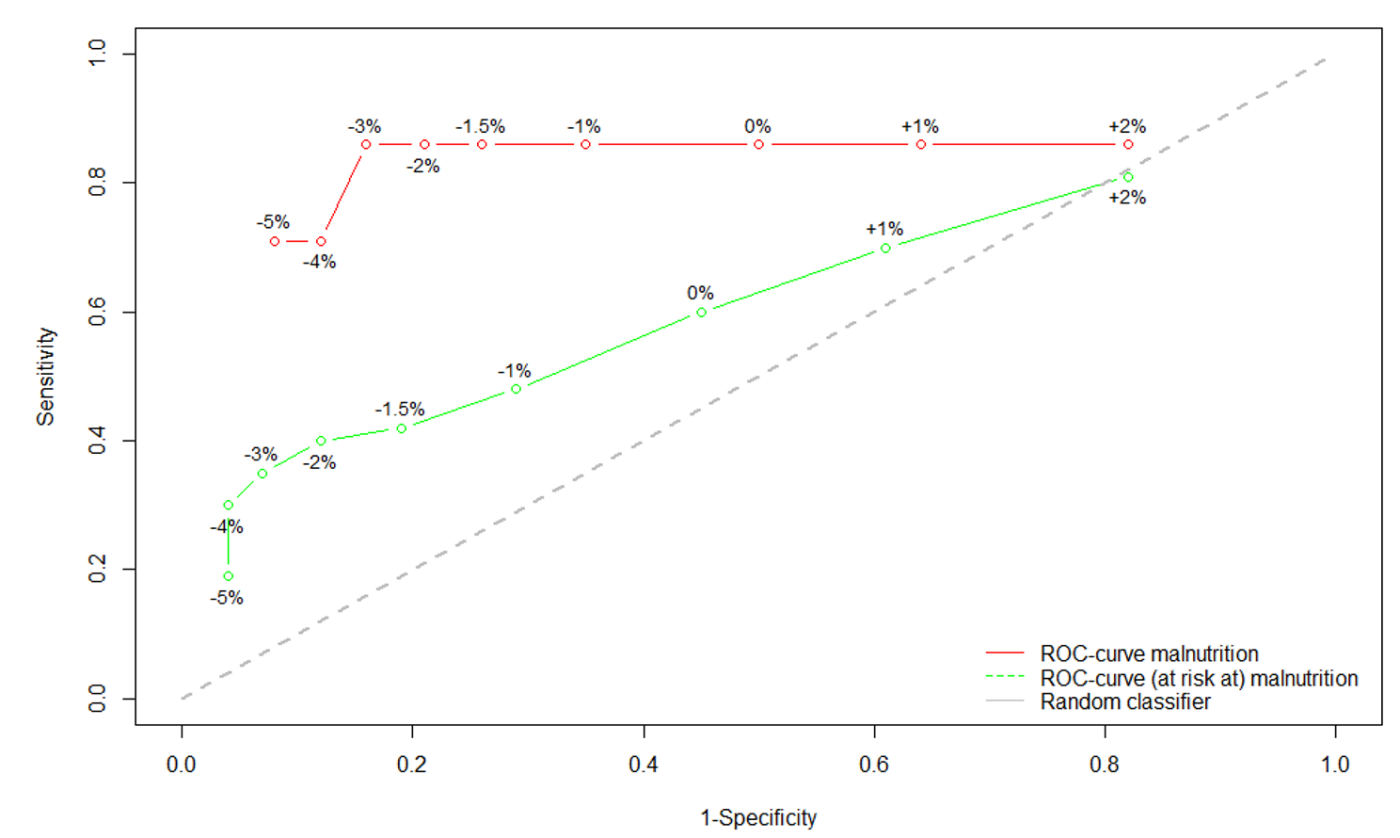
ROC-curves showing the use of evolution of weight in detecting (the risk at) malnutrition (green), and for detecting malnutrition (red). De cut-off values are the difference of the weight evolution (in percentage) between the first and the last weight measurement

In order to detect people with (risk of) malnutrition, this study showed a PPV of 78,6%, a NPV of 60,7%, a sensitivity of 19.3% and a specificity of 96.0% for a weight evolution of ≥ 5% weight loss over six months. To detect malnutrition, our results showed respectively 33,3%, 98,4%, 71.4% and 92.3%. (see Table 2; Fig. 1).

**Table 2:** Calculated positive predictive value (PPV), negative predictive value (NPV), sensitivity and specificity per cut-off value in screening of (risk at) malnutrition (green ROC-curve in Fig. 1) or malnutrition (red ROC-curve in Fig. 1)

	(Risk at) malnutrition				Malnutrition			
Weight loss	PPV	NPV	Sensitivity	Specificity	PPV	NPV	Sensitivity	Specificity
≥ 5%	78,57%	60,68%	19,30%	95,95%	33,33%	98,35%	71,43%	92,25%
≥ 4%	85,71%	63,96%	29,80%	95,95%	23,81%	98,96%	71,43%	87,60%
≥ 3%	80,77%	64,55%	35,00%	93,42%	23,08%	99,09%	85,71%	84,50%
≥ 2%	72,73%	65,05%	40,00%	88,16%	18,18%	99,03%	85,71%	79,07%
≥ 1,5%	64,10%	63,92%	42,11%	81,08%	15,38%	98,97%	85,71%	74,42%
≥ 1%	56,86%	63,53%	48,33%	71,05%	11,76%	98,82%	85,71%	65,12%
≥ 0%	51,43%	63,64%	60,00%	55,26%	8,57%	98,48%	85,71%	50,39%

## Discussion

These results show that weight evolution in people over 70 living at home is a poor predictor in screening for (risk of) malnutrition. Compared to a validated screening tool, the MNA-SF, a weight loss of 5% or more appears to have a sensitivity of only 20% in detecting (risk of) malnutrition. However, a high specificity is observed (96.0%). A possible explanation for the low sensitivity is that the reduction in food intake is already present before weight loss occurs. Severe weight loss would occur too late to function as a nutritional marker in the screening for (risk of) malnutrition in clinical practice [15]. However, weight loss does seem to be a better predictor for detecting malnutrition, with a sensitivity and specificity of respectively 71.4% and 92.3% for a weight loss of 5% or more in this study group.

In this study, the prevalence of malnutrition is 5% and 38% of the participants are at risk of malnutrition. In 2013, the Nutri Action II study reported a prevalence of 7% and 28% respectively among 819 older persons living at home in Belgium [2]. The higher prevalence can partly be explained by the aging of the population accompanied by the accumulation of chronic conditions and the improved survival rates of certain conditions [1].

When developing the MNA-SF as a screening tool, a sensitivity and specificity of 79.5% and 77.4% were described for a BMI < 23 kg/m² as compared to the results on the full MNA [9]. BMI can help to estimate nutritional status but is less accurate as a screening tool than the MNA-SF, which has a sensitivity of 97.8% and a specificity of 94.3% [9].

Our results show that 70.2% (33/47) of people with psychiatric disease have (risk of) malnutrition. This observation supports the finding that the prevalence of (risk of) malnutrition appears to be higher in older persons with dementia or depression as compared to other co-morbidities [2].

A strength of this study is that the predetermined sample size was achieved. In addition, due to the multidisciplinary design and the size of the study population, many different healthcare providers are involved in this study. Another strength of the study is the use of the MNA-SF, a validated and well-developed screening tool for malnutrition for older adults [9]. In addition, patients who were diagnosed with a (risk of) malnutrition during the study were referred to their own GP for treatment and follow up.

A limitation of the study is that we included the home nurses via a convenience sample without randomization. In addition, mainly patients from rural areas were included in this study. Home nurses reported that patients in cities were more reluctant to give their informed consent. This may affect the representativeness of the study population. Furthermore, there sample of home nurses was very diverse, which might compromise the homogeneity. A selection bias is possible when nurses did not complete the study for all their patients who met the inclusion criteria or because certain patient groups refused to participate. The nurses might have deliberately selected patients based on their expected nutritional status, or presented the study only to the more cooperative patient, resulting in a lower prevalence of (risk of) malnutrition. The inclusion of people with dementia might also complicate the assessment of certain questions. Finally, the results referring to the malnutrition category in this study should be interpreted with caution, as the sample size was calculated on the basis of the prevalence of (risk of) malnutrition.

## Conclusion

Weight evolution has a low sensitivity in screening for (risk of) malnutrition in people over 70 living at home as compared to the MNA-SF. In order to detect people with malnutrition, weight loss seems to have a considerable predictive value in this study.

To reliably define sensitivity and specificity of weight loss in the screening of malnutrition, a larger population is required with a sample size calculated on the basis of the prevalence of malnutrition.

Since the MNA-SF is considered to be the best screening tool for malnutrition in community-dwelling older adults, it is interesting to make this screening tool more widely known among primary care providers. This way, the focus will shift from only monitoring weight evolution to a better, more comprehensive screening. resulting in fewer false negatives. A study into how the MNA-SF can be efficiently implemented in primary care, whether or not multidisciplinary, could provide an added value.

## Electronic supplementary material

Below is the link to the electronic supplementary material.

Supplementary Material 1

## Data Availability

The datasets generated and/or analysed during the current study are available in the following repository. https://kuleuven-my.sharepoint.com/:x:/g/personal/birgitte_schoenmakers_kuleuven_be/EcwqUp3juBNLozb74-mWgJIBMp5Urb8eSeXnRjMdj6XGsQ?e=oQ2djQ.
