# Supplementary material for: The predictive value of weight evolution in screening for malnutrition in community-dwelling older persons (70+) in Antwerp
Source: BMC Prim Care. 2023 Mar 6;24:64. doi: 10.1186/s12875-023-02020-w (PMC9987120; doi:10.1186/s12875-023-02020-w)
Supplement: Supplementary file 1 — Supplementary Material 1 [file 12875_2023_2020_MOESM1_ESM.pdf]

ANNEXES The predictive value of weight evolution in screening for malnutrition in community-dwelling older persons (70+) in Antwerp

ANNEX 1 – QUESTIONNAIRE

Overview of data per patient

Last name, first name		
Date of birth		
Gender	M/F	
Telephone number		
Municipality		
Social	Living alone / cohabiting with .... /other, namely	
Home nurse (team)		
General practitioner		
Medication list (possibly in attachment)		
Co-/multimorbidity (known diseases, medical history)		
Date of receipt of informed consent (attach the researcher's copy)		
Weight 1 + date		
Weight 2 + date		
Weight 3 + date		
Weight 4 + date		
Weight 5 + date		
Weight 6 + date		
MNA-SF score + date		

Surname, first name :		Date of birth:
Weight: kg	Length: cm	Date of completion:

**Have you started eating less in the past 3 months due to a decreased appetite, digestive problems, problems with chewing and / or swallowing?**<br>0 = greatly reduced appetite<br>1 = moderately decreased appetite<br>2 = no decreased appetite	
**Weight loss over the past 3 months**<br>0 = weight loss greater than 3 kg<br>1 = don't know<br>2 = weight loss between 1 and 3 kg<br>3 = no weight loss	
**Mobility**<br>0 = tied to bed or chair<br>1 = able to get out of bed/chair independently, but does not go outside<br>2 = goes outside independently	
**Have you suffered from psychological stress or a serious illness during the past 3 months?**<br>0 = yes<br>2 = no	
**Neuropsychological problems**<br>0 = severe demented or depressed<br>1 = mild dementia<br>2 = no psychological problems	
**Body Mass Index (BMI): (weight in kg)/(length in m)²**<br>0 = BMI less than 19<br>1 = BMI between 19 and 21<br>2 = BMI between 21 and 23<br>3 = BMI 23 or more	**If BMI is not known: calf circumference in cm:**<br>0 = calf circumference less than 31 cm<br>3 = calf circumference greater than 31 cm
**Do you think you eat well?**	
**Do you still enjoy eating moments?**	
**Did situations arise during the past 6 months that suddenly changed your weight? And if so, what caused it?**	

ANNEX 2 – MANUAL FOR NURSES FOR CONDUCTING THE QUESTIONNAIRE

Researcher: Evelien Van Offenwert, General Practitioner-in-training

Information for nurses: timeline

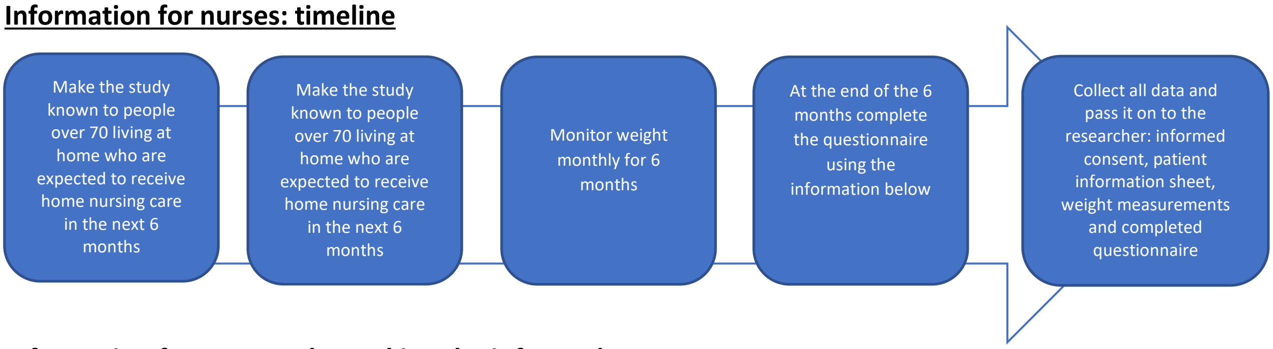

Information for nurses when taking the informed consent

1. **Go over the research question and objectives of the study**
   With this study we want to look at how predictive weight evolution is in the detection of malnutrition in elderly people living at home (70+), compared to the results of a questionnaire that detects malnutrition.
2. **Go over the course of the study**
   The weights will be monitored monthly and recorded for 6 months. At the end of this period, a questionnaire will also be administered that detects malnutrition and gauges the subjective experience around nutrition. This will take 5-10 minutes.
3. **Discuss the possibility of not participating or leaving the study**
   The patient decides for himself/herself whether he/she wants to participate in the study. Participation is voluntary. The patient does not have to say why he/she does not want to participate.
   If the patient does participate in the study, he/she can always change his/her mind and still stop, even during the study. There is no need to give a reason for this.
4. **Discuss the anonymity of data processing**
   The data collected in the context of participation are confidential. Anonymity upon publication is assured.
5. **Emphasize the progress of regular care**
   The patient's usual concerns are continued as usual. The study will have no impact on the normal health care that the patient receives.
6. **Have the patient read the informed consent documents.**
   The complete document is delivered to the patient and he/she must be given sufficient time to read everything thoroughly.
   This concerns the following documents:
   - Necessary information for your decision to participate
   - Informed consent
   - Additional information
7. **Are there any additional questions?**
   If yes, refer to the researcher. The researcher can be reached at her telephone number, or by e-mail (already communicated).
8. **If the patient has had sufficient time to think about this and has been able to ask all his/her questions, the informed consent can still be reviewed and if the patient wishes to participate in the study, it can be signed in duplicate. <u>Please note: the patient must also indicate 2x whether he / she agrees that the doctor is contacted and whether they want to be informed about the results.</u>**

Information for nurses when taking the questionnaire (MNA-SF)[1]

<table>
<tr><td>

Have you started eating less in the past 3 months due to a decreased appetite, digestive problems, problems chewing and / or swallowing?

Question to the patient:

- *"Have you eaten less than usual in the last 3 months?" A: no = 2*
- *If so, "Is this because of a decreased appetite, or chewing or swallowing problems?" A: yes = 0/1, A: no = 2*
- *If so, "Have you eaten much less than before, or just a little less?" A: a lot = 0, a bit = 1*

</td></tr>
<tr><td>

Weight loss over the past few months

- *"Have you lost weight in the past 3 months without making an effort?"*
- *"Is the band of your pants or skirt looser than usual?"*
- *"How much weight do you think you have lost? More or less than 3 kg?"*

Depending on the answers, indicate 0, 1, 2 or 3

</td></tr>
<tr><td>

Mobility

"How would you describe your current mobility?"

- *"Can you get out of a bed, chair or wheelchair on your own without the help of others?" A: no = 0*
- *"Can you get out of a bed or chair on your own, but can't you leave home on your own?" A: yes = 1*
- *"Can you leave the house on your own?" A: yes = 2*

</td></tr>
<tr><td>

Have you suffered from psychological stress or a serious illness during the past 3 months?

- *"Have you been stressed lately?"*
- *"Have you been ill recently?"*

If the answer to at least 1 question is yes, then score 0, if answer no to both questions, then fill in score 2.

</td></tr>
<tr><td>

Neuropsychological problems

- *"Do you have dementia?"*
- *"Have you experienced severe or prolonged sadness?"*

Estimation of home nursing in the degree of neuropsychological problems where score 0 is severely demented or depressed, score 1 mild dementia and score 2 no psychological problems.

</td></tr>
<tr><td>

Body Mass Index (BMI)

BMI = (weight in kg)/(length in m)² - note: measure weight in the morning without clothing, measure height with a measuring rod against a flat wall barefoot if possible.

If not possible, the length can also be measured using the demispan (see image): this is the distance from the midline of the sternal notch to the webspace between the middle and ring

</td></tr>
</table>

fingers with a fully extended arm. In females, the length = (1.35 x demispan in cm) + 60.1. In males, the length = (1.40 x demispan in cm) + 57.8

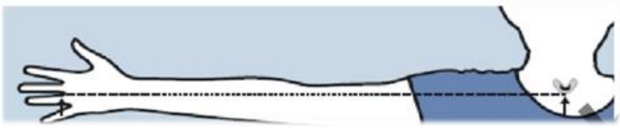
Figure 1

- *0 = BMI less than 19*
- *1 = BMI between 19 and 21*
- *2 = BMI between 21 and 23*
- *3 = BMI 23 or more*

If BMI is not known or length cannot be measured: calf circumference in cm:

Measure calf circumference by having the patient sit with the left leg hanging loose or standing completely upright with the weight evenly distributed over both feet. Roll up the trouser leg so that the entire calf is visible. Put the ribbon gauge around the widest part of the calf and measure there. Also above or below this place make some measurements to be sure that the widest part was measured. Pay attention to the correct angle of the tape measure with the calf length (see image)

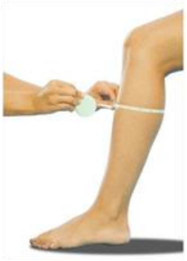
Figure 2 [2]

- 0 = calf circumference less than 31 cm
- 3 = calf circumference greater than 31 cm

Additional question to the patient, preferably answered as openly as possible: "Do you think you eat well?"

Additional question to the patient, preferably answered as openly as possible: "Do you still enjoy eating moments?"

Additional question to the patient, preferably answered as openly as possible: "Did situations arise during the past 6 months that suddenly changed your weight? And if so, what caused this?"

[1] Based on 'A guide to completing the Mini Nutritional Assessment – Short Form (MNA-SF). Consulted via https://www.mna-elderly.com/forms/mna_guide_english_sf.pdf. Consulted on 01/08/2020.
[2] Figures 1 and 2 are from 'Handbook for Nutritional Assessment through Life Cycle'. Retrieved on 04/08/2020 via https://www.researchgate.net/publication/298315075_Handbook_for_Nutritional_Assessment_through_Life_Cycle/figures?lo=1.
